# Duration and determinants of birth interval in Yazd, Iran: a population study

**Published:** 2013-05

**Authors:** Hossein Fallahzadeh, Zohreh Farajpour, Zahra Emam

**Affiliations:** *Department of Biostatistics and Epidemiology, Prevention and Epidemiology of Non-Comunicable Disease Reaearch Center, Health School, Shahid Sadoughi University of Medical Sciences, Yazd, Iran.*

**Keywords:** *Birth intervals*, *Family planning*, *Women*, *Iran*, *Yazd*

## Abstract

**Background: **Short birth intervals have been associated with adverse health outcomes, including infant, child and maternal mortality.

**Objective:** We aimed to investigate the duration and determinants of inter birth intervals among women of reproductive age in Yazd, Iran.

**Materials and Methods: **A cluster sampling technique was used to select 400 ever-married women aged 15-49 years in Yazd, Islamic Republic of Iran. Data were obtained by interview questionnaire and analyzed with life table, Kaplan-Meier survival and Cox regression analyses.

**Results:** The mean duration of inter birth interval was 49.76 (standard error 1.82) months (95% CI: 46.19-53.34 months) with a median of 39. In 28.5% of women the birth interval was <2 years, in 28% it was 3-5 years and in 25.5% it was ≥6 years. Among explanatory variables of interest, age of marriage, and woman's education were significant predicators of the birth interval. Women who stated an ideal preference of two children constituted 59.9% of the sample and 16% had 3 children as well as 10.7% had an ideal preference for 4 or 5.

**Conclusion:** The study recommended an educational program to have optimal birth intervals and ideal number of children per family for the prevention of adverse prenatal outcomes.

## Introduction

Short birth intervals have been associated with adverse health outcomes, including infant, child and maternal mortality. Research has shown that inter-pregnancy interval is a risk factor for pre-term delivery and neonatal death (1). "Short birth intervals may lead to maternal depletion syndrome, milk diminution and competition between siblings close in age for food and other resources" (2). An optimal birth interval, which is the interval between a child and its immediate older or younger sibling, has not been agreed upon universally. Regarding to before studies, for the prevention of the adverse prenatal outcomes the best interval between births is 18-23 months (3, 4). While according to Clayton the optimal interval to ensure survival through childhood is 3 years and 9 months (5). 

From her study in Singapore, Martin deduced that for the best physical and mental development, a minimum of 2 years is necessary between births and if linear trends shown in her results are projected, three years between births would be even better. This might be simply speculative or perhaps a good guess (6). In rural Saudi Arabia, Al-Nahedh and Bella showed that there were significant relation between socio demographic variables and birth interval (7, 8). Two associations have been described in the literature; firstly, that shorter proceeding birth intervals cause increased risk of child mortality, and secondly that child death leads to a subsequent shorter birth interval (9). Results of a Demographic Health Survey study and a meta-analysis showed that previous birth intervals of 36-59 months are optimal for reducing risk of neonatal mortality, although some studies have found significant relation only with shorter spacing childs (10-12). 

Evidence on maternal health has been provided by research managed on over a million pregnancies in 19 countries by the Latin American Center for Perinatology and Human Development. It has observed that birth intervals beyond 2 years (27-32 months) improved maternal health in terms of fewer likelihoods of developing toxemia, anemia and third trimester bleeding as well as 2.5 times less risk of maternal mortality compared to birth intervals of 9-14 months. Intervals longer than 69 months were associated with increased risk of maternal death (10%), third trimester bleeding (10%), eclampsia (80%) and post-partum hemorrhage (90%) (13).

These determinants are the behavioral and biological mechanisms by which fertility is reduced below its biological maximum. Four proximate determinants have been identified: marriage, postpartum in fecund ability, contraception and induced abortion. Social factors such as women’s education, employment opportunities and the number and the sex of surviving children also play a role in determining child spacing (14-16).

In Iran, Hajian *et al *showed that there were significant correlation between birth interval with maternal age, duration of breast feeding, sex of index child, history of still births, history of infant mortality of the index child, type of contraception used, regular attendance at a family planning clinics and parity (17). Other study by Fallahian *et al *found the duration of breastfeeding and the method of contraceptive used were factors significantly associated with child intervals (18). Not only is there a lack of data on the birth interval in Iran, little is known about the perception of Iranian women regarding to ideal preference of children. This study, therefore, aimed to identify the duration and determinants of inter birth intervals among women of reproductive age in the city of Yazd.

## Materials and methods

This descriptive and cross sectional study was conducted in Yazd Province during the period from May 2008 to February 2009.Yazd, one of the large cities of the Islamic Republic of Iran, is the center of Yazd Province. The city is located 750 km south of Tehran. From previous studies, it is known that the maximum SD for birth intervals is 25 and with a marginal of error 3 (17, 18). Thus, a total of 250 women were needed. As cluster sampling was used, the sample size was multiplied by 1.5 (design effect), so 400 cases for a sample size were needed. Sampling was conducted based on the cluster method. In this study we selected 25 homes for each cluster in different sections of the city. Sampling frame used was based on the household lists available in health department in Yazd Province. The cluster number was selected systematically. At the first stage, the number of households for each section was cumulated, and then the sampling interval was computed. 

A random number between 1 and sampling interval was selected. The household in the area corresponding to this selected number was the starting point for first cluster selected. Each succeeding sampling followed the same procedure. The target population of this research was ever-married women of reproductive age. In this study eligibility criteria were being ever married only once and having at least 1 live birth. The inclusion of only women who contributed with at least 1 interval was inevitable since we concentrated on the interval between 2 most recent consecutive live births. Those who were married more than once were excluded to avoid heterogeneity of inter birth intervals for the same woman. 

Data on birth interval were collected using a specially designed questionnaire. Information was elicited from the women by two of trained interviewers. Birth interval was defined as the time period between 2 most recent consecutive births. Socio demographic data were gathered including birthdates of each woman and her husband, education, husband’s occupation, woman’s work status, and family income. Marriage data included date of marriage and current marital status.

Reproductive history included number of pregnancies, dates of pregnancy terminations, pregnancy outcomes including number, status and gender of children, unwanted pregnancy and breastfeeding practices. Women’s opinion of ideal number of children per family was surveyed.

**Statistical analysis**

Data analysis was performed with *SPSS*, version 15. Data were presented as the mean, median and the standard error (SE). Birth intervals determinants were analyzed with Kaplan-Meier survival, Cox regression analyses and the corresponding 95% confidence interval (CI) for Hazard ratio. Significance of results was judged at the 5% level.

## Results

Totally 408 eligible women aged 18-49 years were selected for the study and as 8 of them were not volunteered for entering in the study, finally, 400 eligible women were surveyed. Therefore, the response rate in the study was very high (98%). The mean age was 32.04 (SD=7.01) years. The mean age at marriage was 18.09 (SD=3.38) years with a median of 18 years. The number of children each woman had ranged from 1-7 with an average of 2.17 children per woman [2.17 (SD=1.25)]. On average, women had their first child after 25 months of marriage [25.06 (SD=17.87) months]. The mean duration of the inter birth interval was 49.76 (SE=1.82) months (95% CI: 46.19-53.34 months) with a median of 39. Only 28.3% had a birth interval <2 years, and 25% had a birth interval ≥6 years. Table I show that mean and median inter birth intervals according to woman’s education, husband’s education, woman’s work status and income. With increasing women's education level the birth interval significantly decreased (p=0.0001).

Table 2 shows that 31.5% of intervals were for women aged less than 30 years and the majority of intervals (26.3%) were for women aged 40-50 years. The length of the inter birth interval was not significantly affected by the woman’s age (p=0.437) and the duration of her marriage (p=0.268) at the beginning of the interval. The shortest intervals were those starting with the woman’s age between 40 and 50 years (45.61 months) and in which the woman was married for greater than 20 years (47.38 months). The likelihood of a new live birth was higher for intervals that began between the ages of 30 and 34 years (hazard ratio 0.51, 95% CI: 0.37-0.68) and for those that began between the ages of 35 and 39 years (hazard ratio 0.5, 95% CI: 0.37-0.68). Similarly, the likelihood of a new live birth was significantly lower for intervals that started after 15 years of marriage (Table II).

With regard to table II there was a significant decrease in the inter birth interval with the increase in the age of marriage (p<0.0001). Intervals starting with age of marriage ≤20 years were the biggest (55.12 months). The risk of termination of the interval by a subsequent live birth significantly increased with the age of marriage 20-24 years (hazard ratio 1.6, 95% CI: 1.27-2.02) or age of marriage ≥25 years (hazard ratio 2.3, 95% CI:1.45-3.69). Intervals in which women experienced unwanted pregnancy extended for 47.49 months compared with 50.57 months for intervals in which women did not experience an unwanted pregnancy (p=0.435) (Table III).

Overall 13.5% of the women stated an ideal preference of 1 for the number of children. Women who stated an ideal preference of 2 children constituted 59.9% of the sample and 16% had an ideal preference for 3 children as well as 10.7% had an ideal preference for 4 or 5. Nearly 58% of women had the number of the children who matched their ideal preference. Just 28.5% of the women had not reached their ideal preference, while 23% had exceeded their ideal preference. The mean of the inter birth interval for those who reported an ideal preference of 2 for the number of children or less than 2 was 51.77 (SE=2.53) months compared with 47.52 (SE=1.83) for those who reported an ideal spacing of more 2 for the number of children. This difference was not statistically significant (p=0.178). Cox regression analysis indicated that longer inter birth interval was independently predicted by shorter age of the marriage and lower Woman's education (Table IV).

**Table I:** Mean and median duration of inter birth interval according to sociodemographic characteristics of the women at the beginning of the interval in Yazd in 2009

**Socio demographic characteristic**		**Number (%)**	**Mean±SE** **(months)**	**Median**	**p-value** ^*^	**Hazard ratio** **(95% CI)**
Woman's education						
	Elementary	75 (18.8%)	61.68 ± 4.79	52	0.0001	1
	Guidance	104 (26%)	57.21 ± 4.10	46		1.11 (0.82-1.49)
	High school	161 (40.3%)	42.95 ± 2.34	35		1.67 (1.26-2.21)
	University	60 (15%)	40.22 ± 3.78	30		1.8 (1.28-2.55)
Husband's education						
	Elementary	63 (15.8%)	60.69 ± 5.47	52	0.08	1
	Guidance	108 (27 %)	50.17 ± 3.60	40		1.28 (0.94-1.76)
	High school	159 (39.8%)	46.02 ± 2.72	36		1.45 (1.08-1.94)
	University	706 (17.5%)	47.81 ± 3.70	44		1.4 (0.99-1.98)
Woman's work status (At the interval)						
	Not working	382 (95.5%)	50.36 ± 1.88	40	0.064	1
	Working	18 (4.5%)	36.96 ± 5.41	31		1.55 (0.96-2.5)
Income						
	Low	111 (27.8%)	52.42 ± 3.91	42	0.378	1
	Middle	253 (63.3%)	49.51 ± 2.16	40		1.1 (0.87-1.37)
	High	36 (9%)	43.27 ± 5.62	29		1.43 (0.89-1.89)

* log rank test

**Table II:** Mean and median duration of inter birth interval according to age, duration of marriage number of children at interval onset and years preceding the survey in Yazd in 2009

**Characteristic at interval onset**		**Number (%)**	**Mean** **±** **SE** **(months)**	**Median**	**p-value**	**Hazard ratio** **(95% CI)**
Maternal age (years)						
	18-24	48 (12%)	53.37 ± 4.99	44	0.437	1
	25-29	78 (19.5%)	55.07 ± 4.40	44		0.93 (0.66-1.35)
	30-34	96 (24%)	48.13 ± 4.18	37		1.11 (0.78 – 1.56)
	35-39	73 (18.3%)	49.80 ± 4.12	37		1.09(0.77 -1.73)
	40-50	105 (26.3%)	45.61 ± 3.06	34		1.23 (0.87-1.73)
Duration of marriage (years)						
	1-4	34(8.5%)	60.72 ± 6.45	46	0.268	1
	5-9	84(21%)	51.45 ± 3.94	42		1.23 (0.82-1.84)
	10-14	75(18.8%)	51.58 ± 5.14	37		1.19 (0.79-1.80)
	15-19	72(18%)	45.18 ± 3.68	35		1.49 (0.99-2.24)
	20-39	135(33.8%)	47.38 ± 2.88	35		1.39 (0.95-2.03)
Number of children						
	1	100 (25%)	53.56 ± 3.73	44	0.655	1
	2	141 (35.3%)	48.78 ± 3.36	37		1.13 (0.87-1.50)
	3	60 (15%)	47.18 ± 4.24	36		1.20 (0.87-1.65)
	≥4	99 (24.8%)	48.88 ± 3.23	40		1.14 (0.86-1.51)
Age of marriage (years)						
	≤20	227(69.3%)	55.12 ± 2.31	45	0.0001	1
	20-24	104(26%)	39.05 ± 2.74	29		1.6 (1.27-2.02)
	≥25	19(4.8%)	30.31 ± 5.38	22		2.3 (1.45-3.69)

* log rank test

**Table III:** Duration of inter birth interval in relation to the proximate determinants of fertility at the beginning of the interval in Yazd in 2009

**Variable**		**Number (%)**	**Mean** **±** **SE**	**Median**	**p-value**	**Hazard ratio** **(95% CI)**
Unwanted pregnancy						
	Yes	105 (26.3%)	47.49 ± 3.38	38	0.435^1^	1
	No	295 (73.8%)	50.57 ± 2.13	40		1.5 (1.19-1.92)
Ideal preference for the number of children						
	≤2	241 (60.3%)	51.77 ± 2.53	40	0.178^1^	1
	>2	153 (38.3%)	47.52 ± 1.83	38		1.15 (0.93-1.41)
Sex of child						
	Boy	213 (46.8%)	48.75 ± 2.78	37	0.68^1^	1
	Girl	187 (53.2%)	50.65 ± 2.39	41		1.04 (0.85-1.23)
Breastfeeding						
	Yes	366 (91.5%)	49.74 ± 1.91	42	0.837^1^	1
	No	34 (8.5%)	48.81 ± 6.02	38		1.03 (0.72-1.49)

* log rank test

**Table IV:** Variable predictors of the duration of the inter birth interval in Yazd in 2009

**Variable predictor**		**Coefficient**	**Hazard ratio**	**95% CI**	**p-value**
Age of marriage (years)					
	≤20	Base			
	20-24	0.44	1.56	1.23-1.98	0.0001
	≥25	0.73	2.08	1.23-3.42	0.004
Woman's education					
	Elementary	Base			
	Guidance	0.193	1.22	0.89-1.65	0.212
	High school	0.548	1.73	1.30-2.29	0.0001
	University	0.469	1.6	1.12-2.27	0.009

* Cox regression

## Discussion

This paper looked at the duration of the interval between births and the factors determinant's in Yazd city, Iran. The duration between 2 most recent consecutive live births was of a median duration of 39 Months, which was greater than 3 years, however it is higher than the mean child spacing period of 27 and 26 months found for births of Saudi Arabian children born more than a decade ago (8, 19). This difference is possibly due to a changing secular trend of increasing birth intervals that are occurring in most countries of the world (20). According to information from 55 countries, median birth interval in developing countries was about 32 months (21). In the present study, the birth interval in the majority of the samples was between the recommended range (3-5 yrs) set by the Ministry of Health for Iran. Health education message have focused not only on small family size but also on longer spacing between births (14). 

The median birth-to-conception interval among women in less developed countries who breastfeed their infants is approximately three years (21). Therefore in this study; women whose ideals conformed to these family planning norms had fewer children and longer child spacing intervals. Our study showed there was not a significant association between birth interval and maternal age. 

Decreasing birth intervals, with increasing maternal age, are probably due to different physiological characteristics of different age groups and decreasing fertility with aging. Higher education level is usually linked to better health awareness and longer birth intervals (7, 22, 23). The present study has demonstrated women with higher educational level have shorter birth interval. Similar results have been reported by Al-Nahedh and Hemochandra *et al* (7, 24). In Iran almost all women marry before the end of their child bearing period and the legal age of marriage is 18 years (25). Since child bearing has biological limits, delay in the age of marriage is associated with fewer children per woman (26, 27). 

In present study the mean age of marriage was 18 years which means that the period to bear children was reduced. The present study showed there was not significant association between birth interval and number of surviving children. However other studies have reported that women with low parity had short birth interval (14, 16, 19, 28). Previous studies reported that the sex of children is an influential factor on birth spacing, especially in society's preference for sons dominates (28). 

However in present study, there was not statistical difference between birth intervals in two sexes. In this study, breastfeeding increased the birth interval by an average of 1 month. Independent of other proximate determinants of fertility, breast feeding for 12 months reduced fertility by more than half by increasing the period of postpartum non susceptibility (28-31). 

In the present study, there was significant association between birth interval and marriage age similar to that reported by Mohammadi-Baghmalaie in southern Iran which reported that with increasing marriage age, birth interval decreased (32). This may be because with increasing maternal age and concern of infertility and the occurrence of congenital diseases, the couples decide to have their children soon after marriage. The limitation of the study is that, the effect of birth interval on next birth outcomes was not addressed in the study which needs a prospective study. Random sampling and adequate sample size are the strength of the study. 

The median age of marriage in the center of Iran was lower than the national median of 23.5 years. The finding of the present study cannot be generalized to the whole Iranian community; the main difference is marriage age that has been increased in the recent years. There are two reasons for lower marriage age from national median marriage age. Firstly, the subjects of this study were 15-49 years old group, which some of them had been married many years ago when the marriage age was lower than now. Second, there is a religious belief in Yazd that daughters had better marriage opportunity after finishing the high school.

In our study age of marriage and woman's education were significantly associated with birth interval. Although child spacing in the majority of women was optimal as recommended by the Ministry of Health of Iran (3-5 years), a quarter of women had a prolonged birth interval (≥6 years) which increased the risk for a pregnancy in women age >35 years old. We recommend an educational program to prevent birth intervals beyond the optimal range which increases the risk during pregnancy.
